# Activity and behavior patterns of cattle, horses, and sheep grazing in mountainous areas using geolocation collars

**DOI:** 10.1093/jas/skaf318

**Published:** 2025-09-17

**Authors:** Roger Vidal-Cardos, Emma Fàbrega, Antoni Dalmau

**Affiliations:** IRTA, Animal Welfare, Monells, Catalonia, Spain; IRTA, Animal Welfare, Monells, Catalonia, Spain; IRTA, Animal Welfare, Monells, Catalonia, Spain

**Keywords:** animal activity, extensive production, geolocation collars, grazing patterns

## Abstract

The sustainability of extensive livestock systems is compromised. It is necessary to enhance our understanding of the activity and grazing behavior of different livestock species (cows, horses, and sheep) sharing the same mountainous areas. Nowadays, the observation and analysis of animal activity is greatly facilitated by remote tracking technology, especially in zones with difficult access. In this article, we proved that commercial geolocation collars can provide meaningful data on animal activity, behavior, and distribution, which can be used to model daily distances, activity patterns, grazing behavior, daily home range, and herd dispersal. Results revealed significant differences in activity between species, influenced by the season, altitude, and shepherding practices. Sheep traveled longer daily distances (2.85 km/d) and grazed at higher altitudes than cattle (1.68 km/d) and horses (1.65 km/d), aligning with their specific dietary requirements. Seasonal transhumance and summer conditions also influenced grazing patterns, with peak activity in June and higher altitudes in summer. Cows exhibited a bimodal daily activity pattern, while horses and sheep grazed more consistently throughout the day. Herd dispersal varied by species and season, with cows and horses less dispersed early in the grazing season due to abundant resources. Weather had minimal daily impact, though drier springs in 2022 and 2023 led to increased distances and home range sizes across all species, reflecting stress to find food. Individual variability accounted for much of the observed differences, underscoring the importance of considering individual-specific behaviors in grazing management. These findings highlight the need for species- and herd-customized strategies to promote sustainable livestock management in mountainous rangelands.

## Teaser

This study demonstrates how geolocation collars reveal key differences in grazing behavior, activity patterns, and herd distribution among cows, horses, and sheep in mountainous rangelands.

## Introduction

Extensive livestock production faces critical challenges due to pasture degradation, habitat loss, invasive plant species, and the impacts of global climate change. Consequently, there is increasing interest in detailed, explicit research to identify the factors influencing livestock distribution, behavior, and preferences in extensive farm systems (Robinson et al., 2014; Schroeder et al., 2014). A comprehensive understanding of livestock activity patterns and foraging behaviors within free-range systems is essential for effective management (Senft et al., 1987; Launchbaugh and Howery, 2005). This need is particularly pronounced in large, topographically diverse landscapes where multiple livestock species coexist. These species often repeat distribution patterns seasonally, favoring areas with abundant, palatable forage (Gillet, 2008) and candidates to be overgrazed.

Numerous studies have highlighted factors significantly affecting animal activity and behavior, such as weather and pasture conditions (Bailey, 2005; Hunt et al., 2007; Caton and Olson, 2016). However, few have compared these influences across different livestock species sharing the same grazing area. Understanding these dynamics is vital for developing management strategies that enhance productivity, improve animal performance, and mitigate the environmental impacts of overgrazing (Kairis et al., 2015). Progress in this area is hindered by a lack of multispecies, interseasonal, intensive, and continuous monitoring of livestock mobility and behavior. Monitoring is particularly challenging in vast, remote mountainous regions due to logistical constraints and harsh environmental conditions. Nevertheless, advances in positional technologies now enable the long-term collection of near-real-time spatiotemporal data on animal movements (Ungar et al., 2005).

Global Navigation Satellite Systems **(GNSS)** have been widely adopted to study livestock movements. While visual observations are often limited, GNSS tracking devices facilitate near-continuous monitoring of animals (Shamoun-Baranes et al., 2012). These devices have been utilized extensively in research on grazing systems, particularly for cattle (Agouridis et al., 2004; Knight et al., 2018) and sheep (Putfarken et al., 2008; Fogarty et al., 2014). The positional and temporal data collected through GNSS have contributed to the field of movement ecology, which seeks to understand the drivers, timing, and spatial dynamics of animal movement (Nathan et al., 2008). From GNSS-derived geolocations, movement metrics such as velocity (Laube and Purves, 2011), daily travel distances (Wario et al., 2016), movement direction (Butt et al., 2009), and spatial density (Moritz et al., 2010) can be calculated. These metrics allow researchers to infer behavioral patterns (Patterson et al., 2008; Fryxell et al., 2008; Shamoun-Baranes et al., 2012; Bouten et al., 2013).

Movement behavior changes can now be explicitly quantified using these metrics, linking specific parameters to animal activities (Johnson et al., 2002). For instance, insights into foraging ecology and habitat use can be obtained by pairing location data with activity data (Graham, 2001). Beyond grazing, other activities of interest include traveling and resting. These activities can be inferred from movement velocities: low velocities may indicate resting, moderate velocities grazing, and high velocities traveling (Trotter et al., 2010; Cheleuitte-Nieves et al., 2020). This approach provides a robust framework for understanding and managing the diverse behaviors of livestock in extensive systems.

Livestock movements and their related activity may be influenced by landscape orography and various environmental factors related to seasonality. In paddocks used during the growing season, with higher forage availability and lower grazing pressure (i.e. more available biomass per animal unit), livestock tend to graze for more time and closer (Yu et al., 2024). Conversely, out of the growing season when forage is scarce (and the pressure over resources increases), livestock tend to decrease grazing behavior, travel more, and be more dispersed (Baumont et al., 2000). Therefore, the activities and spatial distribution of livestock are influenced by environmental conditions and processes occurring at different scales, and their activities and distribution can consequently affect patterns of ecosystem structure and function (Goheen et al. 2010). Nonetheless, these kinds of studies are limited by having to use either one species of domesticated animal, enclosed areas, or small sample sizes. Therefore, we aim to describe activity patterns, home range, and animal dispersion over the seasons among three different livestock species (cows, horses, and sheep) grazing together in a natural park. We want to use animal behavior predicted by geolocation data to have a better understanding of grazing interaction in mountainous pastures and demonstrate an effective way to conduct such activity studies with the help of large sample sizes and long-term data collected with geolocation collars, altogether with contextual information such as the orography and weather conditions.

## Material and Methods

All animal procedures used in this study were approved by the Institute of Agrifood Research and Technology **(IRTA)** Ethical Committee, which ensured that the project was adhering to applicable procedures and quality standards in accordance with current legislation (2010/63/EU Directive and 2019/1010 Regulation) and in accordance to the basic Code of Research Ethics proposed in March 2011 by the European Science Foundation **(ESF)** and the European Federation of National Academies of Sciences and Humanities, also known as All European Academies **(ALLEA)**.

### Study area and model species

The study was conducted in the Alt Pirineu Natural Park **(PNAP)**, in the northeast of Spain, located mainly in the region of Pallars Sobirà, Catalonia (42.39′N, 1.19″E) with an extension of 79,317 ha. The PNAP, a mountainous region binding to the Pyrenees, lies between an altitude of 650 m and 3,143 m (Parcs Naturals de Catalunya, 2020). It comprises the axial Pyrenees orographic area, forming a mountain barrier stretching from Vall d’Aran (West) to Andorra (East) for some 30 km. The climate of the area is strongly conditioned by the orography, which can be classified as a Mediterranean-Western Pyrenean type. The annual precipitation ranges from 700 mm at the bottom of the valleys to more than 1,000 mm at the headwaters. Snow is present for approximately 6 mo a year in the higher areas (from November to May). The average annual temperature fluctuates from 3.1 °C to 20.3 °C. The winters are very cold, and temperatures can fall below 0 °C, and the summers can be cool or warm, although temperatures may well reach 30 °C (METEOCAT, 2024).

The PNAP presents a very well-preserved ecosystem of great ecological value, made up of an agroforestry mosaic of mountain forests, scrub areas with pasture, and rocky areas. Most of the territory is covered by Eurosiberian or Boreoalpine vegetation. Below 2,200 m, there are the mountain pine (*Pinus uncinata*) forests; the dominant shrubs are *Juniperus communis*, *Vaccinium myrtillus*, and *Rhododendron ferrugineum*. The grassland is classified as a xerophilic, open montane grassland “Xerobromion” (Carreras et al., 1983), dominated by grasses (*Festuca ovina* and *Festucagautieri*) that co-occur with small forbs (such as *Hieracium pilosella*, *Achillea millefolium*, and *Potentilla neumanniana*). The succession from grassland to shrubland is mostly driven by the dwarf shrubs *Arctostaphylos uva-ursi*, *J. communis*, and *Juniperus sabina*. From about 2,200 m, they are covered with *P. uncinata* and *Abies alba* forests, and in the higher areas, the dominant landscape is natural meadows and rock vegetation (Parcs Naturals de Catalunya, 2020). Moreover, soils experience summer drought episodes, resulting in the apparent drying out of the shallow soil layer.

### Livestock monitoring

We used geolocation collars to monitor 140 cows, 50 horses, and 50 sheep. These animals represented subsets of larger populations, including 15 herds of 732 cows, 6 herds of 100 horses, and 3 aggregated flocks of 2,600 sheep. The three livestock species were from 14 different farmers, providing a representative sample of the whole natural park (Figure S1). The breeds were Bruna dels Pirineus for cows, Pirinenc Català for horses, and Ripollesa and Aranesa for sheep, all local breeds registered in the official book. The animals were monitored with commercial geolocation collars provided by Digitanimal Ltd. (Madrid, Spain) during the grazing season (May to October) for 4 yr (2020–2023). The animals were selected on the advice of the farmers to be representative of the herds’ behavior. The tracking collar system included the following components: a geolocation unit, a lithium battery pack lasting approximately 1 yr, an IP67 enclosure resistant to water and dust, and a low-power wide-area **(LPWA)** communications module based on SigFox, which enabled the transmission of location fixes from collars to the server in near real-time. Geolocation collars were configured to gather a location fix of each animal every 30 min, if SigFox coverage was available, to preserve battery life.

### Characterization of orography and environmental factors

**Figure 1.**
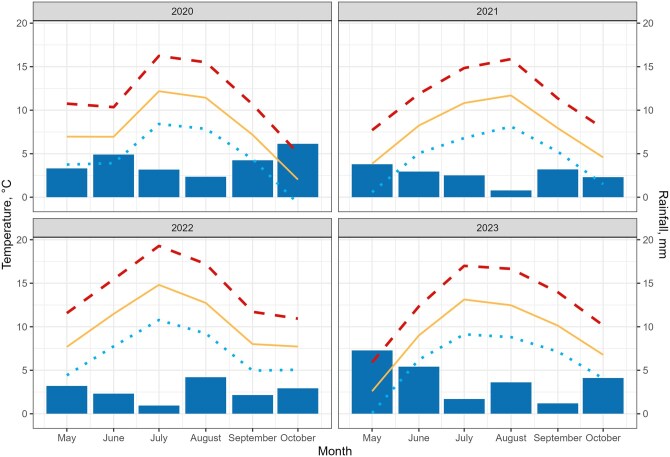
Monthly mean temperatures and rainfall during the grazing period of the 4 yr from three different weather stations at 2,200–2,400 m of altitude. The dashed red line indicates maximum temperatures, the yellow line average temperatures, the blue dotted line minimum temperatures, and the blue bars rainfall.

Several factors may influence livestock activity and behavior, exhibiting either spatial variability (e.g. altitude, slope, etc.) or temporal variation (e.g. weather, season, etc.). We used the elevation aligned to a raster from the Cartographic and Geologic Institute of Catalonia **(ICGC)** and weather data collected from open data sources from the Meteorological Service of Catalonia **(METEOCAT)**. Weather data was from the records of three different weather stations located in Vall d’Àneu (42.65′N, 0.98″E; 2,266 m), Vall del Cardós (42.70′N, 1.27″E; 2,400 m), and Vall Ferrera (42.52′N, 1.36″E; 2,451 m) valleys. The following variables were registered daily: average, maximum, and minimum temperature (°C), rainfall (mm), average relative humidity (%), and average and maximum wind speed (m/s). Figure 1 shows the main temperature and rainfall values recorded at the three weather stations during the grazing periods over the 4 yr.

### Data processing

Prior to activity calculation, as information provided by commercial geolocation collars may contain some missing values due to the lack of SigFox coverage, raw data were preprocessed according to the following steps: (1) From each day, collars that sent less than 10 messages or had more than 12 consecutive hours without sending a message were excluded from data analysis; (2) daily trajectories were calculated using the package “trajr” (McLean and Skowron Volponi, 2018); (3) trajectories with more than 10 km per day or more than 4 km between two geolocations fixes were rejected as not being natural distances; (4) trajectories were resampled to have location fixes at standardized timestamps (every 30 min starting from midnight), having 48 fixes per animal per day; and (5) individual trajectories were smoothed using a Savitzky–Golay filter with a polynomial order of three and a filter length (window size) of five. The Savitzky–Golay filter was used to smooth time series by replacing each value of the series with a new value obtained from a polynomial fit to a certain number of neighboring points (including the point to be smoothed).

After data processing, the remaining location fixes were used to calculate the daily animal trajectory using the package “trajr” (McLean and Skowron Volponi, 2018), the daily home range **(HR)** size based on 95% minimum convex polygons using the package “adehabitat” (Calenge, 2006), and dispersal distance estimated by the average distance between individuals and herd centroid. Afterwards, several indicators were calculated to study the activity and behavior of animals (Table 1). To calculate civil and nautical twilight times throughout the years of study, we used the package “suncalc” (Thieurmel and Elmarhraoui, 2022) as a day length calculator. Nautical twilight was used to categorize the timing of activity. Nautical twilight is defined as the time when the sun is between 12 and 6 degrees below the horizon. Following the methodology described in previous studies (Trotter et al., 2010; Cheleuitte-Nieves et al., 2020), the average velocity between two consecutive locations was used as an indicator of the type of behavior. On average, these authors found that cattle spend 57.5% of their time resting (including rumination), 40% grazing, and 2.5% walking. Thus, they used percentiles 57.5 and 97.5 for the data distribution of the average velocity between consecutive fixes as thresholds to classify livestock locations, which corresponded to resting (low speed), grazing (medium speed), or walking (high speed). We used these percentiles for cattle, but since other livestock species have different speeds, we used the same percentile, adjusted for the speed of each species studied and the slope of the terrain. Herd dispersal while performing different activities, which was estimated as the average distance between individuals and the herd centroid, is another indicator of animal distribution depending on their behavior and species. It is worth noting that the estimation of herd dispersal requires that the location fixes from all animals are synchronized, which is not the case with commercial devices. Then, as it was done in this study, the calculation and resampling of trajectories at standardized timestamps are needed. All these calculations were made in R 4.4.2 (R Core Team, 2022).

**Table 1.** Description of activity and distribution indicators

Variable	Description
Step length	Length of each step within the trajectory, m
Daily distance	Straight-line distance between the start and end of each daily trajectory, i.e. distance between location fix at 0000 on day_n_ and location fix at 0000 on day_n+1_, km/d
Velocity	The average speed between each pair of consecutive fixes recorded, m/s
Home range	The area of the polygon enclosing 95% of the fixes of a daily distance (trajectory), ha
Dispersal distance	Straight-line distance from each location fix of each animal to herd centroid, m

### Data analysis

We built generalized linear mixed-effects models to test which factors affected daily activity, daily HR, and dispersal distance from the herd using the package “lme4” (Bates et al., 2015) and pairwise comparisons computed with the “emmeans” package (Lenth, 2023). The daily activity and HR were log-transformed to ensure normality of error distributions and, in conjunction with dispersal distance, were used as dependent variables. We included weather conditions, year, season, valley, and species as fixed effects, and collar ID (individual) nested within the herd was included as a random effect. Years were included as a fixed effect because each one of them (2020, 2021, 2022, and 2023) presented specific climatic particularities of interest for the comparisons. Model selection for all analyses was based on a stepwise variable selection using Akaike information criterion **(AIC)**, selecting the model with the lowest AIC (Murtaugh, 2009), using the R package “MuMIn” (Barton, 2016). Finally, a bivariate correlation analysis was used to evaluate the relationship between different environmental data. All statistical analyses were carried out in R 4.4.2 (R Core Team, 2024).

## Results

A total of 3,920,688 location fixes were gathered by the geolocation collars used in this study, averaging 33.42 fixes per animal and day from a maximum of 48 fixes. Data gaps might be due to several reasons (as explained in Material and Methods section). During the data cleaning process, 3,337,872 location fixes remained; thus, 14.87% of the available data were deleted due to the cleaning process of the data.

### Animal activity

**Figure 2.**
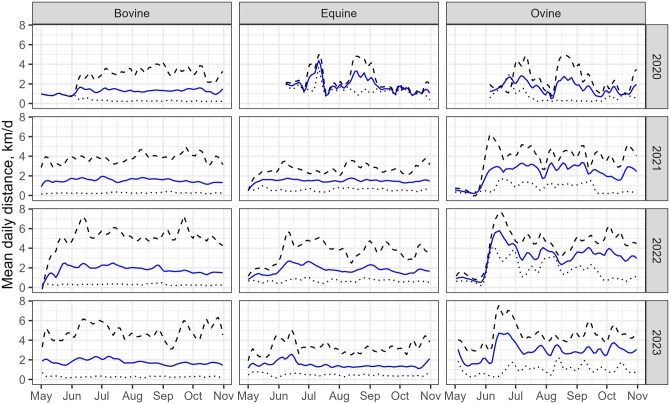
Daily distances covered by cows, horses, and sheep. The blue lines indicate mean daily distances traveled, the dashed lines maximum daily distances, and the dotted lines minimum daily distances covered over the grazing season (May to November) from 2020 to 2023.

To assess the daily distances covered by cows, horses, and sheep, we calculated the mean, maximum, and minimum distances traveled daily over four consecutive years, from May to October (Figure 2). Sheep covered the greatest daily distances, averaging 2.85 ± 1.42 km/d (*n* = 8,960), compared to cows at 1.68 ± 0.96 km/d (*n* = 53,349) and horses at 1.65 ± 1.01 km/d (*n* = 7,230).

The mean daily distance traveled for cows and horses remained relatively consistent throughout the period studied and over the years, though both species showed occasional peaks in maximum distances at certain times of the year. For example, horses increased their movement slightly around mid-June. In contrast, the mean daily distance covered for sheep rose sharply at the beginning of June and fluctuated more irregularly throughout the grazing season. Here, it is important to note that the sheep were managed by a shepherd, so these distances may be biased due to human intervention.

A seasonal effect on the travel distance was also observed in the three species, with higher values in spring and summer compared to autumn (2.01 ± 1.26 km/d, 1.94 ± 1.08 km/d, and 1.64 ± 0.97 km/d, respectively; *P* < 0.05). Additionally, distinct seasonal altitudinal patterns were observed. Sheep grazed generally at higher pastures (mean altitude over the entire period: 1,905.23 ± 333.33 m) compared to horses (1,846.87 ± 400.18 m) and cows (1,775.65 ± 369.22 m), and all livestock species occupied higher altitudes during summer than in spring or autumn (Table 2). These differences may be explained by the specialization of different vegetation types and slopes, which can change markedly over relatively short vertical distances. All these effects explained 21.65% of the variation in daily mean distances, and the random intercept (collars nested within every herd) explained another 15.13%, and herds 17.13% (Table S1).

**Table 2.** Maximum, mean, and minimum daily distances and maximum, mean, and minimum altitudes for cows, horses, and sheep over the seasons

Species	Season	Max dist., km/d	Mean dist., km/d	Min. dist., km/d	Max. altitude, m	Mean. altitude, m	**Min.** **altitude, m**
Bovine	Spring	9.46	1.76 ± 1.22	0.02	2,721.42	1,629.37 ± 365.37*	894.10
	Summer	8.66	1.74 ± 1.03	0.03	2,789.76	1,899.58 ± 356.93*	903.10
	Autumn	9.85	1.47 ± 0.96*	0.01	2,675.21	1,748.48 ± 352.18*	906.26
Equine	Spring	7.49	1.88 ± 1.33*	0.02	2,654.87	1,594.29 ± 426.19*	938.55
	Summer	6.73	1.57 ± 1.04	0.03	2,689.23	2,076.94 ± 302.72*	961.10
	Autumn	6.61	1.47 ± 1.01	0.08	2,648.37	1,848.30 ± 328.93*	939.08
Ovine	Spring	8.90	3.01 ± 2.09*	0.08	2,561.26	1,721.06 ± 389.65*	908.75
	Summer	7.67	2.73 ± 1.31	0.05	2,832.52	2,055.08 ± 229.65*	912.15
	Autumn	8.59	2.63 ± 1.49	0.04	2,648.89	1,830.30 ± 33.73*	884.57

Asterisks indicate statistical significance within the same column and species. *P < 0.05. Satterthwaite type III.

**Figure 3.**
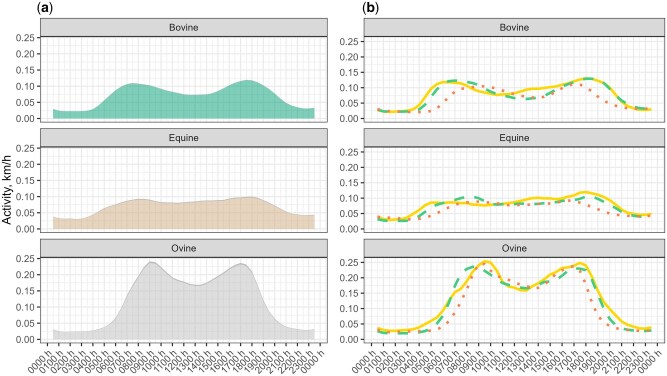
(a) Daily activity profiles from cows, horses, and sheep; (b) daily activity profiles from cows, horses, and sheep in spring (yellow line), summer (green dashed line), and autumn (orange dotted line).

Concerning the timing of movement on a daily basis, animal activity and behavior showed a strong daily temporal component. During the day, there were two main activity peaks, one in the morning and one in the afternoon/evening (Figure 3a). Accordingly, two main resting bouts were also identified, around midday and during the night in the three species, more prominent in cows and sheep. Daily activity patterns were not constant during the experimental period. Significant differences in the average activity at various timestamps were observed between seasons (Figure 3b). The high peak in activity during the morning occurred earlier in spring and summer, while the second peak was delayed around 1 h. Moreover, only in cows and horses, during the spring, there was an increase of activity at midday.

**Figure 4.**
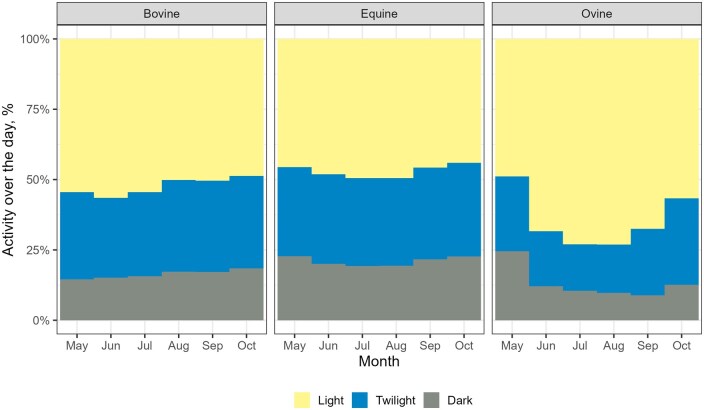
The distribution of activity over the day for each month during the grazing season. Activity distributions were calculated for each species (cows, horses, and sheep). The percentage of activity was calculated as part of the total distance traveled during the 24 h. The yellow portions of the bar represent a fraction of activity during daylight (between sunrise and sunset), the blue portions the fraction during twilight (between nautical twilight and sunrise/sunset), and the gray portion the fraction during the night (between nautical twilight end at dusk and nautical twilight start at dawn).

Due to the pronounced peaks in activity profiles, we plotted the average hourly activity percentages across three distinct parts of the 24-h day: day, night, and twilight. These plots revealed that cows and horses had comparable activity levels, both peaking during daylight hours, while sheep showed even higher activity during the daytime, particularly pronounced in the summer months (Figure 4).

**Figure 5.**
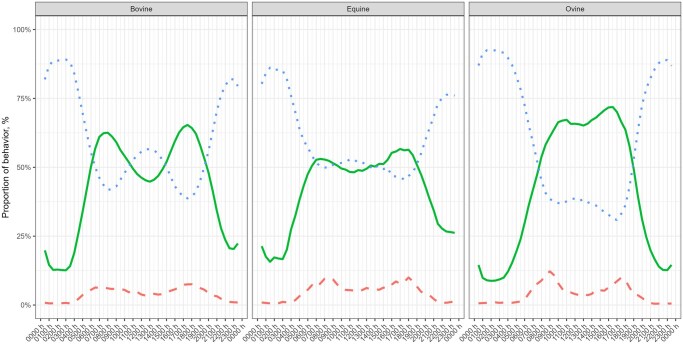
Proportion of grazing (green line), resting (blue dotted line), and walking (red dashed line) during the day in cows, horses, and sheep.

**Figure 6.**
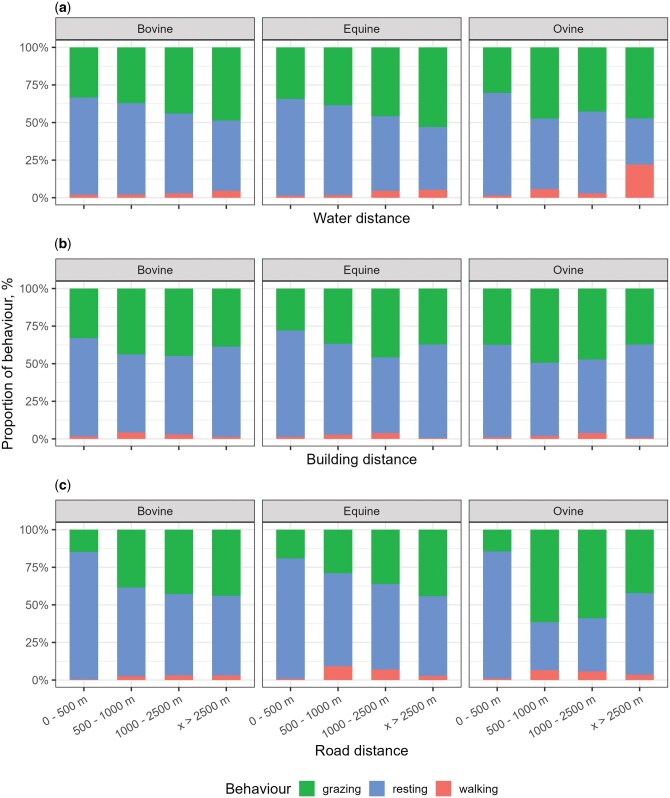
Proportion of grazing (green color), resting (blue color) and walking (red color) depending on the distance between natural water resources (a), buildings (b) and roads (c) from cows, horses and sheep.

While analyzing the different behaviors predicted by the activity of the animals, we found that cows had two clear grazing peaks, one in the morning and another one in the afternoon/evening with a resting peak at midday, while horses and sheep had only one grazing bout during the daylight (Figure 5). In addition, proximity to water resources significantly influenced the animals’ activity patterns and behavior (Figure 6). In particular, cows and horses exhibited increased durations of resting (including rumination) when located closer to water sources. A similar trend was observed with respect to the proximity to anthropogenic structures, such as buildings and roads.

**Figure 7.**
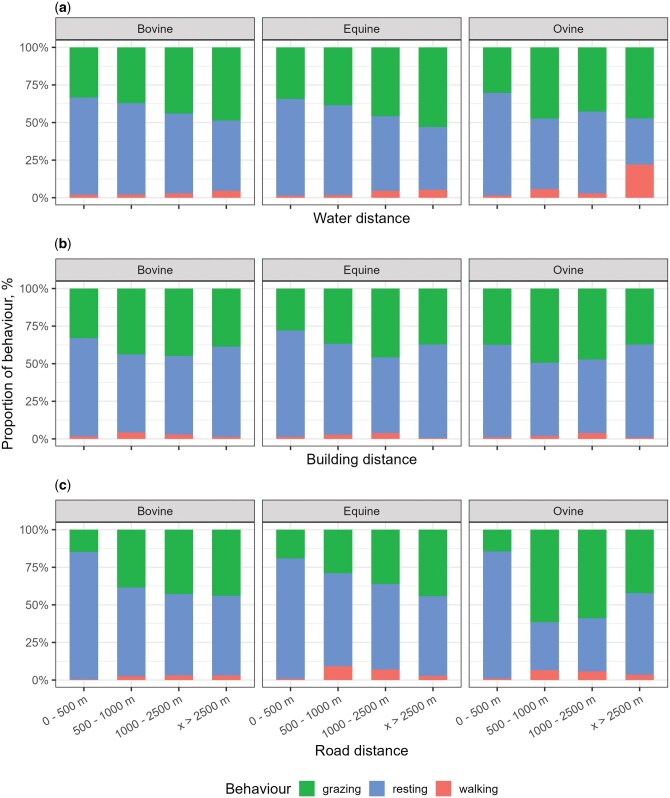
Mean herd dispersal from cows, horses, and sheep over spring (yellow), summer (green), and autumn (orange). Asterisks indicate statistical significance. * = *P* < 0.05.

### Daily home range size

We obtained 68,924 daily HR sizes of 240 individuals during the 4 yr. The cows’ daily HR had a mean of 12.9 ± 17.3 ha and a maximum of 115.45 ha, the daily HR in horses had a mean of 12.1 ± 17.2 ha and a maximum of 92.59 ha, and the daily HR in sheep had a mean of 33.7 ± 31.6 ha and a maximum of 177.21 ha. In addition, daily HR and daily distances were quite correlated (R^2^ adjusted = 0.6391, *P* < 0.05). Sheep had almost three times larger daily HR than cows and horses, and June was the month with the larger daily HR sizes. Cows’ and horses’ daily HR were comparably stable over time, with just a little increase during early summer. Temporal and spatial factors, such as valley, year, mean temperature, relative humidity, and mean wind speed, explained only 13.19% of the total variation in daily HR. The random intercept (individual variability) accounted for another 8.08%, while herds explained 13.79% of the total variability (Table S1).

### Herd dispersal

Cows showed an average herd dispersal of 914 ± 917.63 m (*n* = 51,971), horses 666 ± 866.24 m (*n* = 6,076), and sheep 384 ± 691.78 m (*n* = 8,682). Regarding seasonal effects, the cows and horses were significantly more dispersed in autumn in contrast with sheep (bovine: estimate: 0.97; SE: 0.10; z-ratio: 9.60; *P* < 0.05; equine: estimate: 0.65; SE: 0.14; z-ratio: 4.58; *P* < 0.05) (Figure 7).

In addition, the models only explained 5.45% of the variability of the fixed factors, and random factors such as individuals and herds explained the other 38.48% and 31.26% of variability, respectively (Table S1). This indicates that individual differences played a more significant role in influencing dispersal variation than the environmental and spatial-temporal factors studied. We have also seen a positive correlation between dispersal distance and speed (estimate: 2.29; SE: 0.12; *t*-value: 18.42; *P* < 0.05).

### Environmental effects

To compare different weather parameters, first, we deleted correlated variables, calculating adjusted Pearson correlation coefficients (R^2^) within a matrix. We deleted maximum temperature and minimum temperature since they were strongly correlated to mean temperature (R^2^ = 0.98 and 0.96, respectively) and maximum wind speed because of the strong correlation with mean wind speed (R^2^ = 0.75). In general, in all the variables: daily distance, HR, and herd dispersal, weather conditions had a low or no effect (Table 3 and Table S2). Nonetheless, differences in the climate over the years did have an influence: in the years 2022 and 2023, with higher temperatures and less rain, animals had greater activities, larger daily HR, and longer herd dispersal (Table 4).

**Table 3.** Informative estimates of the fixed effects for the weather analysis of daily distance, home range, and herd dispersal

Daily distance	Estimate	SE	*t*-value	*P*-value
TM	0.015	0.001	8.8	<0.05
HRM	0.001	0.001	15.8	<0.05
PPT	−0.001	0.001	−1.2	0.23
VV10M	−0.007	0.001	−18.3	<0.05
**Daily HR**				
TM	0.045	0.002	27.6	<0.05
HRM	0.004	0.001	12.1	<0.05
PPT	0.001	0.001	0.2	0.84
VV10M	0.001	0.002	0.1	0.87
**Herd dispersal**				
TM	0.005	0.001	6.8	<0.05
HRM	0.001	0.001	4.0	<0.05
PPT	0.001	0.001	2.3	<0.05
VV10M	−0.014	0.002	−6.8	<0.05

**Table 4.** Mean and SE of daily distance, home range, and herd dispersal over the years 2020, 2021, 2022, and 2023

	2020	2021	2022	2023
Daily distances, km/d	1.45 ± 0.88^a^	1.72 ± 1.02^b^	1.97 ± 1.22^c^	1.87 ± 1.08^c^
Daily HR, ha	9.62 ± 13.70^a^	13.20 ± 18.60^b^	17.50 ± 23.90^c^	14.50 ± 20.30^b^
Herd dispersal, m	761.03 ± 819.23^a^	765.59 ± 870.87^a^	725.52 ± 848.29^b^	975.63 ± 990.56^c^

Different superscript letters within the same row indicate statistical significance (*P* < 0.05).

## Discussion

Several authors have pointed out the necessity of understanding livestock activity and grazing behavior in free-range systems to improve grazing management strategies (Laca, 2009; Schnyder et al., 2010; Byambaa and de Vries, 2021). This study proposes several approaches to understanding and quantifying activity patterns using geolocation collars. Unlike previous studies (Bailey et al., 2018; Augustine & Derner, 2013; Polojärvi et al., 2010), research was conducted by combining commercial geolocation devices and open data sources, which allow us to conduct long-term studies in the whole grazing period on the mountains. Although the data presented some limitations due to a lack of coverage at certain times and locations, these limitations could be overcome through specific data processing strategies, which enabled the extraction of meaningful information on animal behavior and distribution in pastures.

In the present study, we have studied and compared the activity of three different livestock species coexisting in the same mountainous area over 4 yr. The mean daily distance traveled by cattle and sheep was shorter (1.68 km/d and 2.85 km/d, respectively) than other data published from other studies in cows (Pauler et al., 2020 [4.1 km/d]; Pérez et al., 2018 [3.3 km/d]) and in sheep (Tsevegemed et al., 2019 [13 ± 1.3 km/d]; Wade et al., 2024 [6–9 ± km/d]). This is probably because they reported cattle and sheep in flatter and smaller rangeland pastures, while the livestock of our study grazed on larger mountainous areas at higher altitudes and with an irregular topography. Sheep had longer mean daily distances and tend to graze at higher elevations than cows and horses. This aligns with previous studies (Dumont et al., 2002; Parsons et al., 1994), which highlight the need for sheep to seek more specific types of food due to their unique fiber tolerance and protein requirements (Jarman, 1979) compared to cows and horses. In consequence, smaller ruminants graze the upper levels of mountain summer ranges and larger ruminants the lower ones. The outcome generates an interesting vertical stratification of animals in relation to the body size (Garcia-Gonzalez et al., 1990). However, this behavior can also be influenced by shepherding practices, as shepherds often tend to guide sheep to graze at higher altitudes. All species displayed a peak of mean distance traveled in June due to the livestock transhumance when herds are driven from winter pastures or stables to summer pastures. That is the reason why in spring or early summer daily mean distances were longer across all three species. Moreover, the three species inhabit at higher altitudes in summer. This aligns with the warmer conditions of the season, likely prompting animals to seek cooler mountain breezes, increase their visits to water sources (Garcia-Gonzalez et al., 1990; Raniolo et al., 2022), and search for fresh, windy places to reduce the effects of heat and escape biting insects (Hughes et al., 1981). In fact, time spent resting and ruminating is higher near natural water sources.

In daily activity patterns, cows and sheep demonstrate the frequently reported bimodal activity pattern (Hart et al., 1993; Schoenbaum et al., 2017) defined by early morning and late afternoon grazing periods with an intermediate resting period at midday. This bimodal pattern of grazing was less evident in horses. In fact, as it has been already recorded (Arnold, 1984; Patkowski et al., 2019), horses spend more time grazing and less resting than cows and sheep during daylight hours. This pattern may be explained by the fact that horses are nonruminants and therefore need to graze for longer and more continuous periods to compensate for their less efficient digestive system (Archer, 1973). Seasonal activity profiles revealed that the activity peaks of both cows and sheep generally followed daylight hours, indicating that day length strongly influenced activity rhythms in temperate climates. Moreover, sheep showed higher activity during daylight in summer, when day length was longest, compared with the other seasons. Nevertheless, in sheep, the two daily activity peaks remained relatively similar across seasons, with the autumn morning peak occurring even earlier than in spring. This contrasts with other studies (Ravault et al., 1999; Wyse et al., 2018; Ikurior et al., 2021), where sheep follow daylight hours more precisely. These discrepancies might be explained because the sheep movements were largely determined by the shepherd, and sheep spend their resting times enclosed in a place near the shepherd’s hut in an open sheepfold. On the other hand, horses did not display a clear daily activity peak across seasons, suggesting that their activity patterns may be less influenced by daylight duration, in line with behavioral and physiological evidence reported in previous studies (Murphy et al., 2011; Ensing et al., 2014).

We found that sheep had three tines the larger daily HR sizes than cows and horses, coinciding with longer daily mean distances. In fact, animals traveled a distance, and HR had a high correlation. Thus, the increase in travel distance is related to the increase of exploration of larger pasture areas. In addition, due to this correlation, temporal and spatial factors affected daily HR and daily distance in the same way.

Regarding herd dispersal, cows were the most dispersed, followed by horses, with sheep being the least dispersed, and the distance between individuals and herd centroids was strongly influenced by the season. As we expected, cows and horses were less dispersed at the beginning of the grazing season (spring) (Halley et al., 2002; Cheleuitte-Nieves et al., 2018, 2020) compared to the end (autumn). This is explained because of the higher amount of food resources on the pasture, which allows animals to be closer without competing with each other. However, in sheep, it was totally the opposite. Again, we believed that how the shepherd drives the sheep biased the results. Interestingly, random effects, i.e. variation between individuals, explained similar or more variation in daily distances, HR, and herd dispersal than the spatial and temporal factors studied. In this way, our results add to the increasing understanding of the relevance of individual differences when studying animal behavior and ecology (Bowler and Benton, 2005; DeAngelis, 2018; Mayer et al., 2018).

Finally, in our study, weather had a minimal impact on animal daily distance, daily HR sizes, and herd dispersal, aligning with some studies (van Beest et al., 2011) yet contrasting with others (Garcia-Gonzalez et al., 1990; Arnold, 1984; Rivrud et al., 2010). These contradictory results may be explained because we recorded mean weather conditions per day, while variation in animal activity could be at a more specific level, like on an hourly basis, since changes in weather are in a short time and often during the whole day. Another explanation could be that we were able to only register temperatures for three weather stations when the weather varies a lot depending on the place and altitude (Baigorria et al., 1999; Beniston, 2006; Hoy et al., 2018). However, there was a notable effect on a broader climatic scale, such as seasonal changes (Mayer et al., 2018) and over the years. In the years 2022 and 2023, which started with a drier spring, all animals in general showed higher distance recorded and a larger daily HR. This seems to point to a major stress to find food resources in the pasture zones, since the quality and quantity of grazing plants depends to a large extent on the amount of rain in the spring.

## Conclusion

The results obtained in our study confirmed, in a large mountain rangeland, the differences among cows, horses, and sheep sharing the same pasture area over time. Sheep traveled longer distances and grazed at higher altitudes than cows and horses, influenced by their dietary needs and shepherding practices. While cows and sheep showed a bimodal activity pattern, horses grazed more consistently throughout the day. The consideration of temporal and spatial scales had a low effect on animal activity compared to interspecific individual and herd variability. While daily weather had minimal impact, broader climatic trends, such as dry springs, affected grazing behavior (e.g. a change in vegetation availability). These findings emphasize the need to consider species, seasonal, and individual factors in animal activity and sustainable grazing management in mountainous areas.

## Supplementary Material

skaf318_Supplementary_Data

## Data Availability

The data presented in this study are available on request from the corresponding author. The data are not publicly available because they mostly refer to animal coordinates, the property of different farmers.

## Abbreviations:

AIC: Akaike information criterion
ALLEA: All European Academies; ESF, European Science Foundation
GNNS: Global Navigation Satellite Systems
HR: home range
ICGC: Cartographic and Geologic Institute of Catalonia
IRTA: Institute of Agrifood Research and Technology
LPWA: low-power wide-area
METEOCAT: Meteorological Service of Catalonia
PNAP: lt Pirineu Natural Park
