# Supplementary material for: Activity and behavior patterns of cattle, horses, and sheep grazing in mountainous areas using geolocation collars
Source: J Anim Sci. 2025 Sep 17;103:skaf318. doi: 10.1093/jas/skaf318 (PMC12558749; doi:10.1093/jas/skaf318)
Supplement: skaf318_Supplementary_Data [file skaf318_supplementary_data.docx]

**Supplementary table 1:** The model selection results for the analysis of the (1) daily distance, (2) daily home range and (3) herd dispersal of livestock showing the full, best, and intercept only model. Collar ID nested within the herd was included as random effect. R^2^m 0 marginal R^2^ and R^2^c = conditional R^2^.

| **Model** | **df** | **LogLik** | **AICc** | **Delta AICc** | **AICc weight** | **R^2^m**  **(individual)** | **R^2^c**  **(individual)** | **R^2^m**  **(herd)** | **R^2^c**  **(herd)** |
| --- | --- | --- | --- | --- | --- | --- | --- | --- | --- |
| 1. **Daily distance** |  |  |  |  |  |  |  |  |  |
| Year + season + valley + sp + altitude + TM + HRM + PPT + VVM10 **+ water point + buildings + roads** | 21 | -83220.2 | 166480.5 | 1 | 0.38 | 0.2173 | 0.3682 | 0.3158 | 0.4872 |
| Year + season + valley + sp + altitude + TM + HRM + VVM10 **+ water point + buildings** | 18 | -83221.5 | 166479.1 | 0 | 0.62 | 0.2165 | 0.3678 | 0.3156 | 0.4869 |
| Intercept only | 3 | -86454.2 | 172914.4 | 0.10 | 0 | 0 | 0.3327 | 0 | 0.2738 |
| 1. **Daily Home Range** |  |  |  |  |  |  |  |  |  |
| Year + season + valley + sp + altitude + TM + HRM + PPT + VVM10 **+ water point + buildings + roads** | 21 | -90133.1 | 180306.3 | 2.28 | 0.13 | 0.1321 | 0.2124 | 0.1785 | 0.3163 |
| Year + season + valley + sp + TM + PPT **+ water point + buildings + roads** | 18 | -90134.8 | 180303.6 | 0 | 0.56 | 0.1319 | 0.2127 | 0.1783 | 0.3162 |
| Intercept only | 3 | -91803.9 | 183613.8 | 0.23 | 0 | 0 | 0.1959 | 0 | 0.2415 |
| 1. **Herd dispersal** |  |  |  |  |  |  |  |  |  |
| Year + season + valley + sp + altitude + TM + HRM + PPT + VVM10 **+ water point + buildings + roads** | 21 | -82707.3 | 165454.6 | 0.77 | 0.26 | 0.0545 | 0.4400 | 0.3975 | 0.7102 |
| Year + season + valley + sp + altitude + VVM10 **+ water point + buildings + roads** | 18 | -82709.7 | 165453.4 | 0 | 0.38 | 0.0545 | 0.4393 | 0.3971 | 0.7097 |
| Intercept only | 3 | -83862.4 | 167730.9 | 1.10 | 0.17 | 0 | 0.4050 | 0 | 0.1580 |

**Supplementary table 2**: Estimate, standard error (SE), z-value and P-value of explanatory variables for the analysis of the (1) daily distance, (2) daily home range and (3) herd dispersal of livestock.

| **Variable** | **Estimate** | **SE** | **t-value** | **P-value** |
| --- | --- | --- | --- | --- |
| 1. **Daily distance** |  |  |  |  |
| Year 2021 | 0.04 | 0.02 | 2.96 | P < 0.05 |
| Year 2022 | 0.23 | 0.02 | 13.92 | P < 0.05 |
| Year 2023 | 0.21 | 0.02 | 13.30 | P < 0.05 |
| Season Summer | -0.11 | 0.01 | -11.44 | P < 0.05 |
| Season Autumn | -0.29 | 0.01 | -32.03 | P < 0.05 |
| Species Equine | 0.01 | 0.08 | 0.03 | P = 0.98 |
| Species Ovine | 0.98 | 0.05 | 20.46 | P < 0.05 |
| Ferrera Valley | -0.41 | 0.05 | -9.10 | P < 0.05 |
| Àneu Valley | -0.17 | 0.04 | -5.04 | P < 0.05 |
| Altitude | -0.01 | 0.01 | -2.09 | P < 0.05 |
| TM | 0.06 | 0.01 | 14.08 | P < 0.05 |
| HRM | 0.02 | 0.01 | 4.24 | P < 0.05 |
| PPT | 0.01 | 0.01 | 1.26 | P = 0.20 |
| VVM10 | -0.01 | 0.01 | -2.56 | P < 0.05 |
| Water points | 0.27 | 0.01 | 53.85 | P < 0.05 |
| Buildings | 0.04 | 0.01 | 6.90 | P < 0.05 |
| Roads | -0.01 | 0.01 | -1.06 | P = 0.29 |
| 1. **Daily home range** |  |  |  |  |
| Year 2021 | 0.03 | 0.02 | 1.74 | P = 0.08 |
| Year 2022 | 0.18 | 0.02 | 10.30 | P < 0.05 |
| Year 2023 | 0.11 | 0.02 | 6.56 | P < 0.05 |
| Season Summer | -0.10 | 0.02 | -9.87 | P < 0.05 |
| Season Autumn | -0.21 | 0.01 | -20.88 | P < 0.05 |
| Species Equine | -0.04 | 0.04 | -1.14 | P = 0.25 |
| Species Ovine | 0.78 | 0.04 | 21.54 | P < 0.05 |
| Ferrera Valley | -0.19 | -0.03 | -5.61 | P < 0.05 |
| Àneu Valley | -0.04 | 0.03 | -1.51 | P = 0.12 |
| Altitude | 0.01 | 0.01 | 1.09 | P = 0.27 |
| TM | 0.04 | 0.01 | 7.04 | P < 0.05 |
| HRM | 0.01 | 0.01 | -1.25 | P = 0.21 |
| PPT | 0.01 | 0.01 | 3.70 | P < 0.05 |
| VVM10 | -0.01 | 0.01 | -0.92 | P = 0.35 |
| Water points | 0.20 | 0.01 | 35.70 | P < 0.05 |
| Buildings | 0.08 | 0.01 | 12.89 | P < 0.05 |
| Roads | -0.04 | 0.01 | -5.02 | P < 0.05 |
| 1. **Herd dispersal** |  |  |  |  |
| Year 2021 | -0.08 | 0.01 | -4.12 | P < 0.05 |
| Year 2022 | -0.09 | 0.01 | -5.1 | P < 0.05 |
| Year 2023 | 0.23 | 0.01 | 13.69 | P < 0.05 |
| Season Summer | 0.06 | 0.07 | 5.84 | P < 0.05 |
| Season Autumn | 0.25 | 0.07 | 27.40 | P < 0.05 |
| Species Equine | -0.26 | 0.02 | -3.04 | P < 0.05 |
| Species Ovine | -0.44 | 0.02 | -5.69 | P < 0.05 |
| Ferrera Valley | 0.14 | 0.01 | 2.12 | P < 0.05 |
| Àneu Valley | 0.18 | 0.01 | 4.26 | P < 0.05 |
| Altitude | 0.04 | 0.01 | 5.35 | P < 0.05 |
| TM | 0.01 | 0.01 | 0.09 | P = 0.93 |
| HRM | 0.01 | 0.01 | -0.01 | P = 0.99 |
| PPT | 0.01 | 0.01 | 2.08 | P < 0.05 |
| VVM10 | -0.01 | 0.02 | -3.63 | P < 0.05 |
| Water points | -0.04 | 0.01 | -8.43 | P < 0.05 |
| Buildings | 0.07 | 0.01 | 12.17 | P < 0.05 |
| Roads | -0.03 | 0.01 | -4.16 | P < 0.05 |


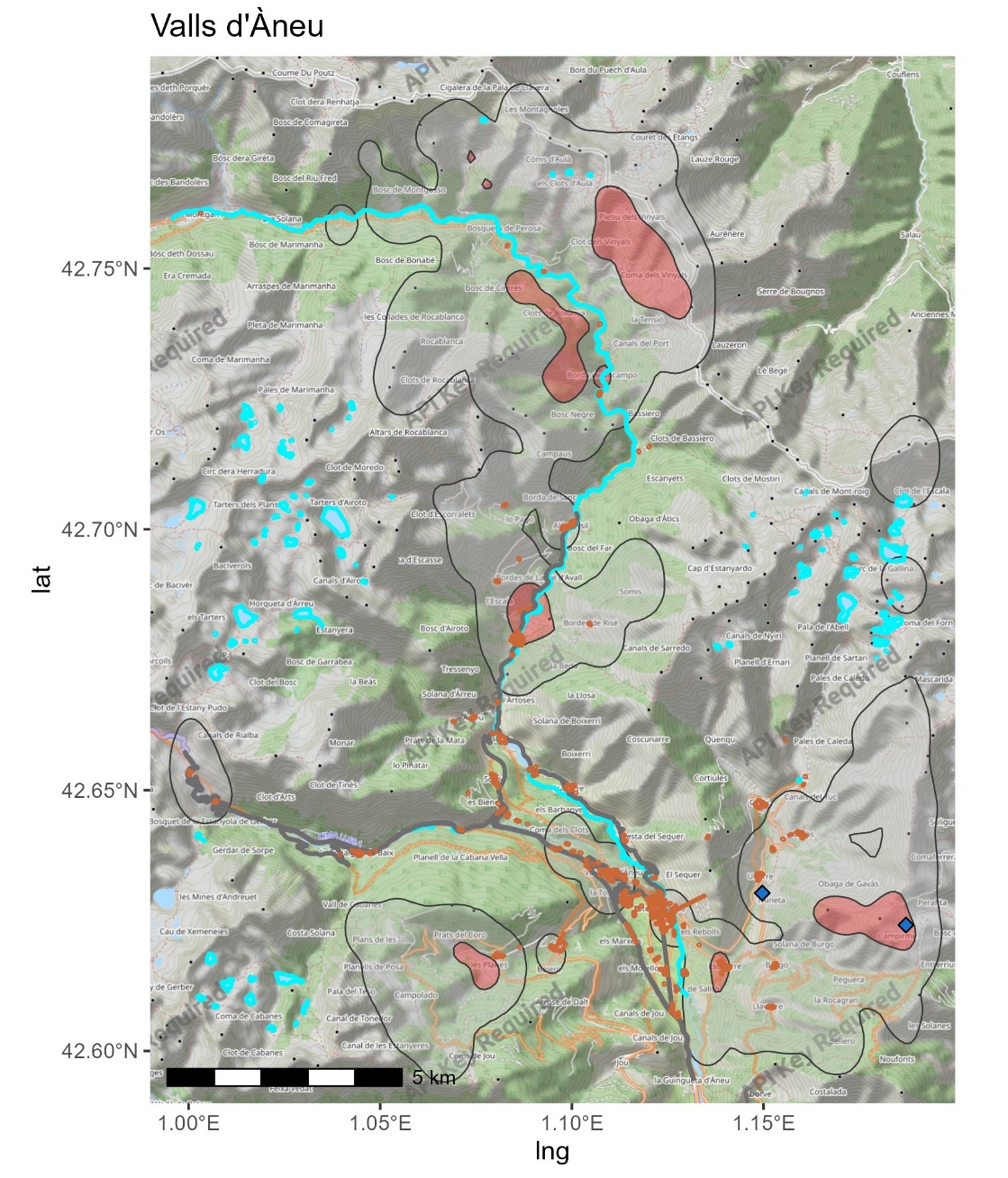

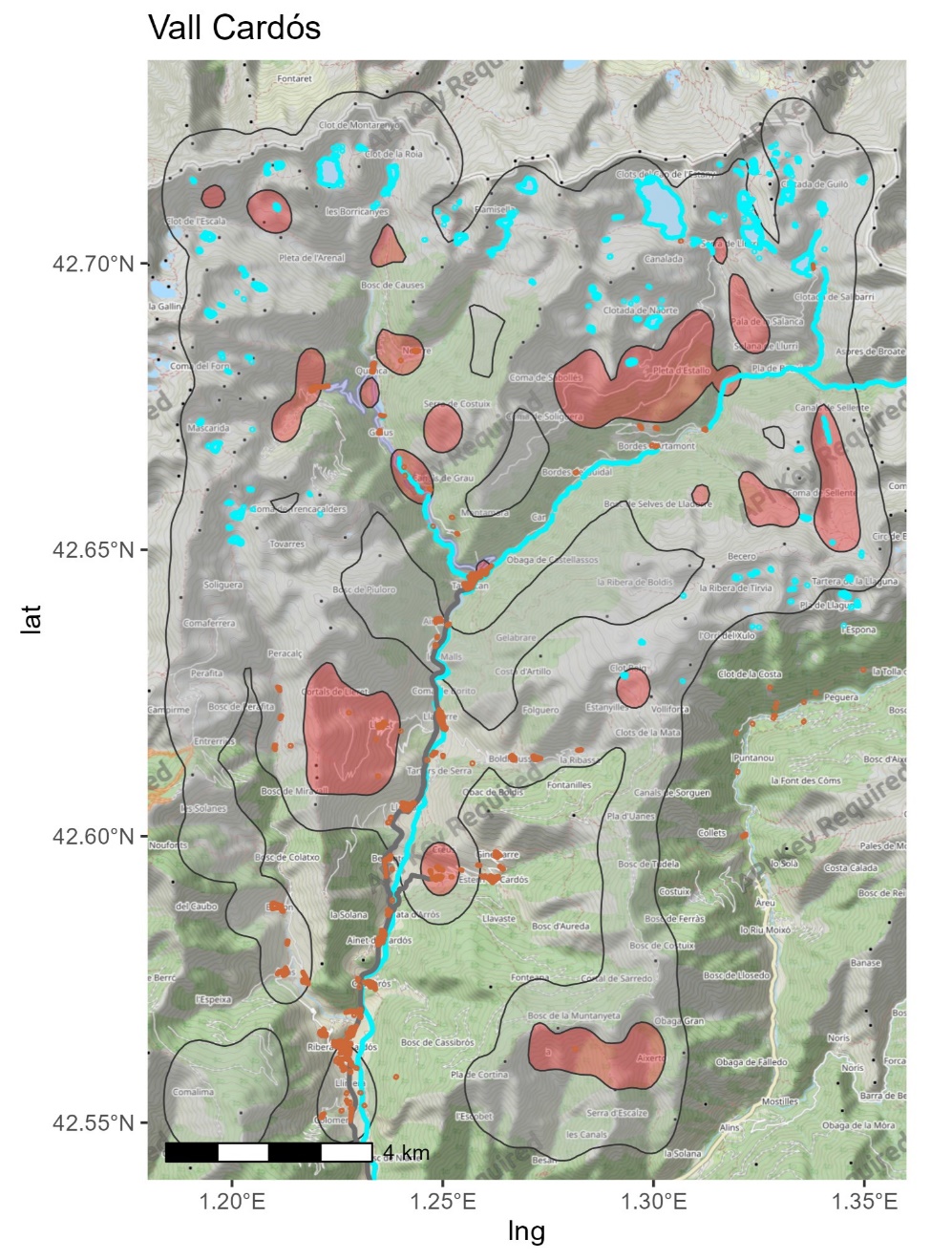


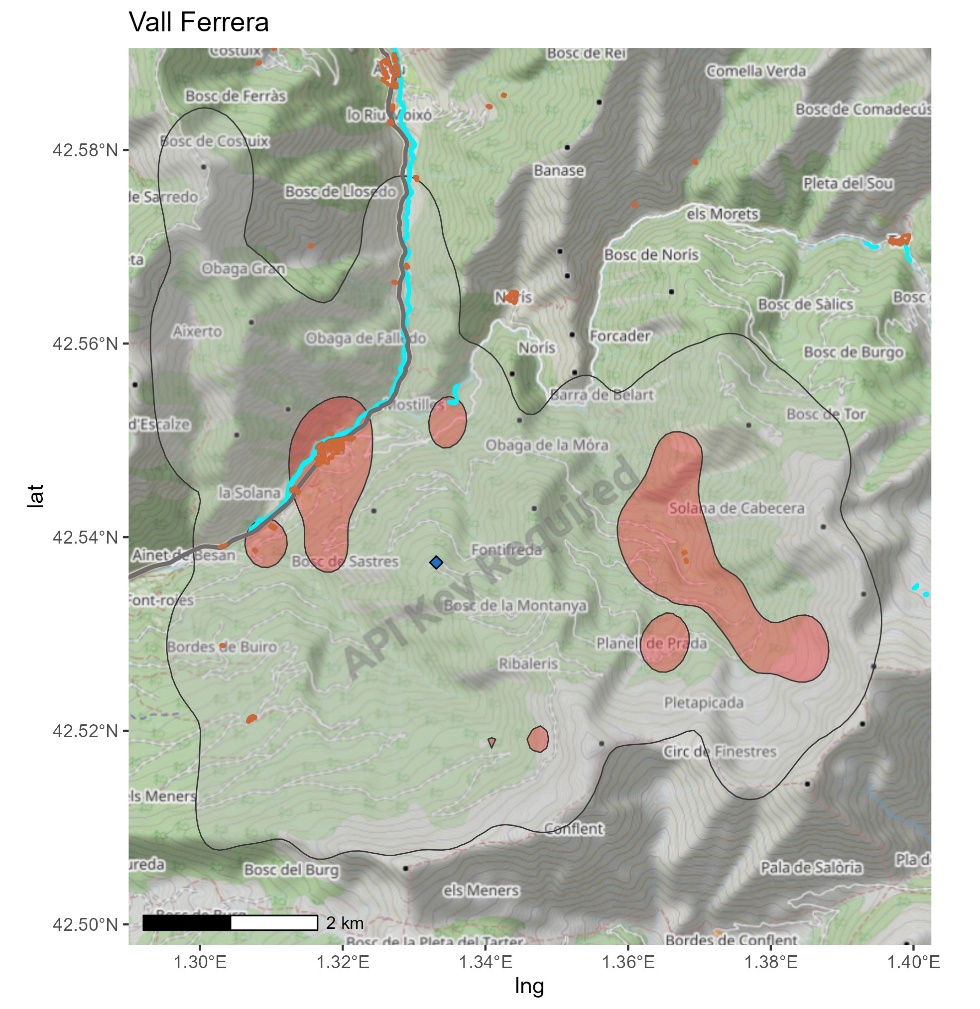


Fig. S1. Maps showing the location of the three study areas (Valls d’Àneu, Vall Cardós, and Vall Ferrera). Red polygons indicate areas with 95% livestock prevalence, while grey polygons represent the entire pasture area. Light blue lines represent rivers and lakes, grey lines denote roads, orange lines show buildings, and blue diamond figures indicate watering points. Please note that each map has a slightly different scale.
